# Suprasellar arachnoid cyst revealed by central diabetes insipidus

**DOI:** 10.11604/pamj.2025.52.71.49392

**Published:** 2025-10-14

**Authors:** Youssra Lammini, Faycal El Guendouz

**Affiliations:** 1 Department of Endocrinology and Metabolism, Moulay Ismail Military Hospital, Meknes Faculty of Medicine, Pharmacy, and Dentistry, Sidi Mohammed Ben Abdellah University, Fez, Morocco

**Keywords:** Central diabetes insipidus, arachnoid cyst, hypothalamic-pituitary MRI

## Image in medicine

We report the case of a 29-year-old man with severe polyuria and polydipsia (~25 litres per day) affecting the quality of the patient's sleep, persistent fatigue, and chronic headaches. Laboratory evaluation revealed hypernatremia (145 mmol/L), elevated plasma osmolality (298.65 mOsm/kg), which contrasts with low urine osmolality (121 mOsm/kg), confirming diabetes insipidus without deficient secretion of anterior pituitary hormones. The desmopressin administration test confirmed the diagnosis of central diabetes insipidus, showing a total response to desmopressin. Magnetic resonance imaging of the hypothalamic-pituitary region revealed an arachnoid cyst with the absence of the posterior pituitary bright spot (Panel A), while the anterior pituitary gland appeared preserved (Panel B). Treatment was conservative with simple desmopressin replacement therapy without surgery. This case illustrates the rare association between an arachnoid cyst and central diabetes insipidus, probably due to chronic hypothalamic compression rather than traction of the pituitary stalk. Prompt recognition and management are crucial for restoring fluid balance, improving sleep, reducing fatigue, and minimising the psychosocial impact. This report highlights the clinical importance of hypothalamic-pituitary MRI in the management of central diabetes insipidus without resorting to the fluid deprivation test, which is poorly tolerated by patients.

**Figure 1:**
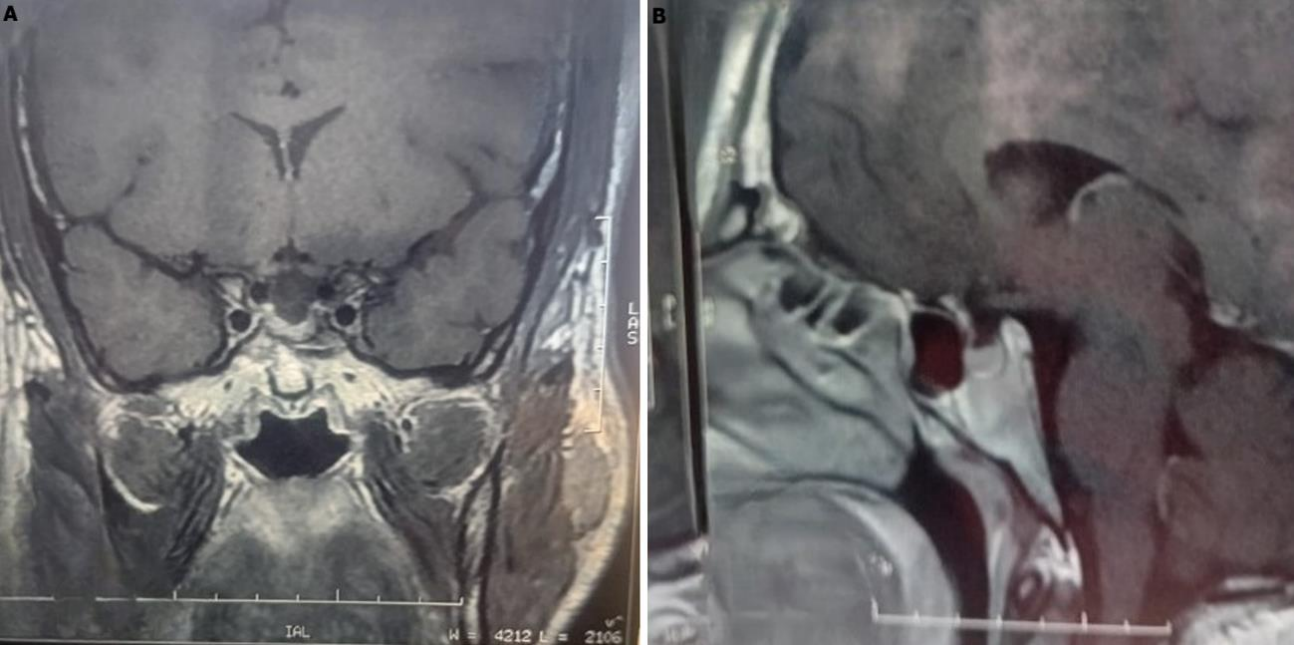
T1-weighted MRI of the hypothalamic-pituitary region (A) coronal; B) suprasellar arachnoid cyst with downward herniation of the suprasellar cistern; the posterior pituitary bright spot is absent, while the anterior pituitary gland is preserved

